# The impact of climate change on mental health

**DOI:** 10.1192/j.eurpsy.2024.29

**Published:** 2024-08-27

**Authors:** A. Heinz

**Affiliations:** Department of Psychiatry and Psychotherapy, Charité - Universitätsmedizin Berlin, Berlin, Germany

## Abstract

**Abstract:**

Climate change has a profound impact on mental health, supported by meta analytic evidence. For every degree temperature increase, there is a statistical increase in mental health problems by about 0.9%. The direct association between catastrophic events such as hurricanes or flooding and traumatization or negative mood states is evident. However, there are also interactions between pollution or heat islands in urban contexts and stress associated mental disorders, and there are indirect interactions e.g. between loss of agricultural space, poverty, displacement and mental health challenges. We provide an overview regarding direct and indirect effects of climate change on mental health and discuss possible interventions on the health care system.

**Disclosure of Interest:**

None Declared

